# Challenges and Attitudes of Clinicians Concerning the Communication of Risk and Lifestyle Advice for Hypertension and Diabetes: A Qualitative Study

**DOI:** 10.7759/cureus.111201

**Published:** 2026-06-20

**Authors:** Kritika Singhal, Smriti Bhaskar, Suruchi Gupta, Alka Modi Asati, Pankaj Prasad

**Affiliations:** 1 Community and Family Medicine, Ram Krishna Medical College Hospital and Research Centre, Bhopal, IND; 2 Community and Family Medicine, All India Institute of Medical Sciences, Bhopal, Bhopal, IND; 3 International Health, Johns Hopkins Bloomberg School of Public Health, Baltimore, USA; 4 Community Medicine, Shyam Shah Medical College, Rewa, IND

**Keywords:** behavioral risk factors, clinicians, consultation, diabetes, health communication, hypertension, lifestyle modification, patient-centered care, patient education and counseling, risk communication

## Abstract

Background: India is witnessing an epidemiological shift from infectious to noncommunicable diseases. Traditional patient education and counseling methods fail to motivate patients to make desirable changes to manage their disease. This study explores clinicians' perceived challenges and their attitudes toward communication practices, particularly risk communication and lifestyle advice for hypertension, diabetes, and related behavioral risk factors.

Methods: In-depth semi-structured interviews were conducted with 14 clinicians, including consultants and residents from the Departments of Cardiology, General Medicine, and Family Medicine at a tertiary care hospital in India. Interviews were audio-recorded, transcribed verbatim, and analyzed using the framework method of thematic analysis. A hybrid deductive-inductive coding approach was employed to identify emerging themes related to clinicians’ perceptions, communication practices, and barriers to risk communication.

Results: Five major themes emerged: perceptions of the burden of noncommunicable diseases, challenges in language and patient understanding, patient-related barriers, clinician communication practices, and awareness of risk communication. Clinicians routinely provided medication and lifestyle advice based on standard guidelines and tailored their communication according to patients’ literacy, age, and socioeconomic background. However, high patient load and limited consultation time restricted detailed communication, resulting in reliance on informal rather than structured risk communication methods. Participants expressed interest in formal training and standardized approaches for communicating disease risk.

Conclusion: Clinicians recognized the importance of communicating advice on disease risk and lifestyle modification but largely relied on informal communication practices rather than structured methods. Time constraints, language barriers, and patient-related factors were perceived as major challenges. The findings suggest a need for developing and evaluating standardized, context-specific risk communication strategies and training programs to strengthen patient-centered care in the management of hypertension and diabetes.

## Introduction

Non-communicable diseases (NCDs), such as cardiovascular diseases (CVDs), hypertension, and diabetes, have emerged as a significant public health burden globally and in India [1]. A meta-analysis in 2025 found the pooled prevalence of CVDs in India to be 11% [2]. CVDs have many risk factors like hypertension, diabetes, tobacco or alcohol consumption, inadequate consumption of fruits and vegetables, physical inactivity, and raised cholesterol. Hypertension and diabetes are major public health challenges in India, affecting nearly one-third and one-fifth of adults, respectively [3,4].

The National Program for Prevention and Control of Noncommunicable Diseases encompasses guidelines for the prevention and control of NCDs, including population-based, opportunistic screening, and behaviour change communication [5]. Despite these efforts, the management of diseases continues to rely predominantly on pharmacological therapy supplemented by patient education. In this context, patient-physician communication is an integral component of clinical care in the long-term management of chronic diseases. Physicians need to establish rapport and trust with patients during consultations to facilitate the exchange of health information, while patients are expected to voluntarily provide details about their medication adherence and lifestyle behaviour for an accurate prognosis for each patient [6]. However, evidence indicates that conventional methods of health education, typically short, non-standardized, and generic, are often ineffective in changing long-term behaviour or improving adherence [7]. This leads us to the fundamental question, "What information will truly make an impact?" Risk communication, therefore, assumes particular importance, as effective communication is integral at all stages of healthcare delivery [8].

Risk communication is recognised as a two-way process involving engagement with affected populations to enable informed decision-making for themselves and their loved ones. It involves interactive exchange of information regarding the probability, severity, and consequences of disease, enabling informed decision-making. Unlike routine counselling and health education, risk communication specifically focuses on communicating uncertainty and future health risks [9]. The adoption of health-protective behaviours is strongly influenced by an individual’s perception of their own health risk, often described as perceived susceptibility [10]. As a structured approach to conveying information about disease probability and severity, risk communication holds promise in enhancing risk perception and promoting effective self-management [11].

One of the primary challenges in risk communication lies in developing and delivering messages that are not only accurate but also meaningful, engaging, and persuasive [12]. Evidence suggests that individuals who are better informed about their cardiovascular risk are more likely to adopt preventive behaviours and demonstrate improved health outcomes [13]. Nevertheless, several factors, including physicians’ communicative style and tone, as well as patients’ education and health literacy, can substantially influence the quality and effectiveness of risk communication, often acting as barriers to the exchange of health information [6].

Although several studies have examined patient education and physician-patient communication, very few studies have explored how Indian clinicians conceptualize and practice risk communication in the routine management of hypertension and diabetes. Existing literature primarily focuses on patient outcomes rather than clinicians’ perspectives, leaving an important gap regarding barriers, attitudes, and implementation challenges within resource-constrained settings. In this context, the present study aims to explore how clinicians perceive risk communication, current communication practices, barriers to effective risk communication, and attitudes toward adopting structured risk communication strategies.

## Materials and methods

This was a qualitative descriptive study conducted in the Department of Cardiology, General Medicine, and Family Medicine at All India Institute of Medical Sciences (AIIMS), Bhopal, Madhya Pradesh, India, from June 2021 to August 2021, to explore clinicians’ perceptions, attitudes, and challenges in communicating with patients with hypertension and diabetes regarding risk and lifestyle modification. The reporting of this qualitative study was guided by the Consolidated Criteria for Reporting Qualitative Research (COREQ) checklist [14]. The study was approved by the Institutional Human Ethics Committee-Post Graduate Research (IHEC-PGR), AIIMS, Bhopal (registration number: IHECPGRMD026).

Study population

A purposive sampling method was employed to recruit clinicians managing patients with NCDs. Inclusion criteria included physicians involved in routine diagnosis and management of hypertension, diabetes, and related risk factors. Non-consenting clinicians were excluded. The study included two consultants and residents from the Cardiology department, two consultants, and three residents each from the General Medicine and Family Medicine departments. 

Data collection

Fourteen in-depth interviews (IDIs) were conducted across a mix of consultants and residents across three departments (Cardiology, General Medicine, and Family Medicine). Interviews were conducted using a guide developed from existing literature and expert consultations (see Appendices). The interviews, lasting 45-60 minutes each, were conducted in person in the clinicians’ offices to ensure comfort and confidentiality. All interviews were audio recorded with participants' consent and transcribed verbatim. No one other than the interviewer and the participant was present during the interviews.

Reflexivity

The interviewer was trained in qualitative interviewing and had no supervisory relationship with participants. Reflexive notes were maintained during coding to minimize personal bias and enhance analytical transparency.

Data Saturation

Data collection and analysis occurred concurrently. Interviews were conducted till saturation, when no new themes emerged in the final two interviews, and thematic categories became repetitive. After the 12th interview, no substantially new codes emerged. Two additional interviews were conducted to confirm thematic redundancy. As no novel concepts were identified, thematic saturation was considered achieved after 14 interviews.

Coding

Initial open coding was performed independently by two researchers. Codes were subsequently grouped into categories and organized into broader themes through iterative discussion using the framework method. Deductive codes originated from the interview guide, whereas inductive codes emerged from repeated reading of transcripts.

Data analysis

Thematic analysis was performed using the framework method. Transcripts were uploaded into RQDA software version 0.3-1 (R package version 0.2-8. http://rqda.r-forge.r-project.org/) for coding. A hybrid coding approach was employed: deductive codes were generated from the interview guide, while inductive codes emerged during line-by-line analysis. The coding scheme was refined iteratively and grouped into three overarching domains: clinician perceptions, communication challenges, and attitudes toward risk communication. Inter-coder reliability was ensured through independent double coding of a subset of transcripts, with discrepancies resolved through discussion.

## Results

The major themes identified from the IDIs were “perceptions of NCD burden”, “challenges in language and understanding”, “patient-related challenges”, “clinician communication practices,” and “risk communication awareness”. Table 1 showcases the emergent themes, subthemes, and representative participant quotations from the thematic analysis.

**Table 1:** Emergent themes, subthemes, and representative participant quotations from the thematic analysis NCD: non-communicable diseases

Main theme	Subthemes	Quotations	Mapped objective	Description
Perceptions of NCD burden	Diseases of concern	"…. concerned about hypertension and diabetes." "considerable effort being put in towards cancer, heart disease, diabetes." "most people who present to us are presenting with symptoms…."	Perception	Clinicians express concern about underdiagnosis and low patient awareness of hypertension and diabetes.
	Incidental discovery		Perception	Many cases are detected during unrelated visits, not due to active symptom recognition.
Challenges in language and understanding	Medical vs local terminology	"different terminologies they use for stroke like 'hawa lag gayi hain (superstitious belief)' and 'dil ki bimari hai (disease of the heart)', 'nase block hai dil ki (heart vessels are blocked)".	Challenge	Clinicians report a communication gap between medical language and patients’ local terms or beliefs.
	Misconceptions		Challenge	Patients often hold culturally ingrained explanations, making biomedical counseling difficult.
Patient-related challenges	Denial of diagnosis	"Initially, most patients show denial reaction…accuse the interpersonal variability when it comes to BP", "They are afraid that they might also suffer something similar because some friend/relative had a heart attack due to blood pressure", "When I tell them that you will have to take the medicines for life, they get scared….are they causing any side effects….how costly the medicine could be."	Challenge	Many patients initially reject the diagnosis, suspecting device errors or downplaying risk.
	Fear of lifelong medication		Challenge	Long-term treatment plans evoke resistance and fear.
Clinician communication practices	Customizing advice	"Prevention of CVD is the main goal", "The new parameter, i.e., HbA1C, also has to be targeted," "If the patient is old or uneducated, we cannot keep stringent goals," "No shortcuts for medication. A patient cannot skip the medicines," "They have more compliance if the drug is not that expensive," "Reducing the number of medications that the patient has to take. It also helps with compliance," "We ask smokers to quit, obese patients to lose weight, alcohol users to stop alcohol, sleep well, and follow general precautions," "Do not use a lift. Park vehicles a little far away to be able to walk the distance," "Should divide their meals into three meals during the day….calorie distribution…," "If you substitute one habit with another, they tend to be more reinforced….instead of the two cigarettes, you should supplement that with physical activity," "Dose of bp medications would be reduced if they exercise regularly," "We are not able to do audio-visuals…we do give them some diet charts," "We do not have staff to do this for us," "I usually draw a diagram of a plate and tell them about the diet," "Enough time to explain the advice during consultation??.........not always, not always," "Once they become used to…..then they do not seek much of our time," "Get more aware with time about their diseases, so they notice small changes, they are more aware of the check-ups," "They start to understand the linkage between the complications and these chronic diseases without any symptoms," "These patients are keen to know what they can do now."	Attitude	Clinicians adapt advice based on patient age, literacy, and socioeconomic status.
	Time constraints		Challenge	High patient load limits the depth of counseling.
Risk communication awareness	Informal practice	"Maybe this term is new to me, or I cannot comprehend it," "Informally, we do risk communication; we do explain to the patient," "It is crucial, but it is neglected…it is very practical," "Communication is a time-consuming process...lack of time and the burden of patients that stops physicians from doing it with every patient," "Many times we (clinicians) are not aware what should be done, kinds of words and what precise information…that sits in patients' brain, and which will make these patients more compliant".	Attitude	Risk communication is acknowledged but practiced without formal methods.
	Training need		Attitude	Clinicians show interest in structured training and medico-legal clarity.

Perceptions of NCD burden

".... concerned about hypertension and diabetes."

"considerable effort being put in towards cancer and heart disease, diabetes."

"most people who present to us are presenting with symptoms...."

"Diseases that affect a large number of populations" concerned the clinicians. Hypertension and diabetes were considered "very concerning" mainly because these were lifestyle diseases and were common nowadays. Patients did not perceive their severity and required more effort. Stroke, myocardial infarction, and other complications like foot ulcers, heart failure, etc., were essential diseases. Apart from NCDs, communicable diseases considered important included tuberculosis, malaria, dengue, HIV-AIDS, and COVID-19. However, clinicians reported that their patients were more concerned about "symptoms more than the disease itself." Symptoms like cold, cough, fever, numbness in legs, body ache, or skin lesions were of more importance to patients.

In regular clinical practice, hypertension, diabetes, and CVD were prevalent. However, they reported that patients were usually unaware of their suffering from any of these three diseases. Most of the time, it was an incidental finding during routine check-ups. Further, modifiable behavioral risk factors like tobacco or alcohol use, unhealthy diet, and physical inactivity were also found in patients. Most common among them were smoking and alcohol use, which were more common in the male gender and lower socioeconomic status. Physical inactivity was common in higher socioeconomic status.

Challenges in language and understanding

"terms like bp badha hua hai aapka (blood pressure is raised), stroke hua hai (he/she has stroke), lakwa pada hai (paralysis), dil ka bimari hai (heart disease) are used for heart diseases."

Patients usually use crude terms like "high BP," "blood pressure," and "*ucch raktchap*" for hypertension. While for diabetes, they use words like "*madhumeh*" and "*shakkar ki bimari*" or even the word "diabetes" itself. CVDs have various terms ranging from "attack," "heart *ka bimari*", "*dil ka bimari*", "*dil ka daura*", "*hawa lagi thi*," and "*dil ki nass *block* hui thi*." Similarly, clinicians also used a mix of medical jargon and crude terms used by patients to explain the diseases and the treatment during consultations.

Patient-related challenges

"Initially, most patients show a denial reaction…accuse the interpersonal variability when it comes to BP."

"They are afraid that they might also suffer something similar because some friend/relative had a heart attack due to blood pressure."

"When I tell them that you will have to take the medicines for life, they get scared….are they causing any side effects….how costly the medicine could be."

Clinicians witness varied concerns of patients regarding their initial diagnosis of hypertension, diabetes, and CVDs. Initial reactions constitute a denial of the diagnosis altogether. They even blame the blood pressure apparatus and glucometers, stating they might have faulty readings. A strong disagreement is usually expressed when the symptoms are not perceived to be severe enough by the patients. They may also fear potential complications or mortality from hypertension and diabetes, as they had seen similar events in their acquaintances. They are apprehensive about the curability of their hypertension, diabetes, or CVDs. They also express distress over the medication's duration, cost, and side effects. Clinicians deal with these reactions by agreeing and reasoning with valid concerns. Clinicians try to explain the diagnosis and treatment on subsequent occasions to make the patient more comfortable with their disease. Sometimes, dire consequences are explained to make them perceive the severity of it.

Customizing advice

"Prevention of CVDs is the main goal."

"If the patient is old or uneducated, we cannot keep stringent goals."

"They have more compliance if the drug is not that expensive."

"Reducing the number of medications that the patient has to take. It also helps with compliance."

"We ask smokers to quit, obese patients to lose weight, alcohol users to stop drinking, sleep well, and follow general precautions."

"Do not use a lift. Park vehicles a little far away to be able to walk the distance."

"Should divide their meals into three meals during the day….calorie distribution."

"If you substitute one habit with another, they tend to be more reinforced… instead of the two cigarettes, you should supplement that with physical activity."

"We are not able to do audio-visuals…we do give them some diet charts."

"I usually draw a diagram of a plate and tell them about the diet."

"Enough time to explain the advice during consultation??.........not always, not always."

"Get more aware over time about their diseases, so they notice small changes, and they are more aware of the check-ups."

"They start to understand the linkage between the complications and these chronic diseases without any symptoms."

Clinicians have specific treatment goals for their patients, and these include medication compliance and lifestyle modification. Furthermore, preventing complications is the final and main treatment goal. Further, clinicians usually keep stricter goals for young patients than elderly patients. Education will determine the understanding of the disease and treatment, and socioeconomic status directly affects the ability to maintain treatment adherence. Lastly, a pre-existing lifestyle leads to a willingness to change, as habits are difficult to change, but slight variations from routine are acceptable.

Clinicians feel that patients have to be continuously motivated for medication. Lowering the medication cost or altering the dosage is done for better compliance. They reinforce the advice by detailing the consequences of discontinuing medication, such as "high chances of stroke, myocardial infarction, or other CVDs." Medication advice is always based on blood glucose and pressure. Sometimes, co-existing diseases like chronic kidney disease also warrant more caution about compliance.

Reducing salt and sugar is the most common lifestyle modification advice given by clinicians for hypertension and diabetes. In addition, incorporating a calorie-based diet, reducing oil consumption, increasing fiber intake, and dividing meals are also advised. They also refer them to dietitians sometimes. Patients are motivated to lose weight by using stairs and walking wherever possible. Yoga and brisk walking are also advised. Whenever required, psychiatrists or psychologists deal with tobacco and alcohol usage.

Furthermore, motivating patients when the family is well-informed about the disease is easier. Another way is to gradually reduce unhealthy habits, as it is difficult to change habits. Sometimes, clinicians suggest a healthier alternative for an unhealthy habit. Clinicians also use blood pressure and glucose values to motivate patients towards healthier habits.

The advice is usually verbal, and sometimes pamphlets are given if available. Clinicians also drew rough diagrams of a balanced plate on the prescription papers. All clinicians reported that they themselves gave medication and lifestyle advice as they did not have any other staff for it. Furthermore, they had insufficient time to give advice properly due to the high patient load. However, they felt that the time required for advice is reduced with follow-ups.

Clinicians feel it is easier to deal with previously diagnosed patients as they know everything from before. They have better follow-ups and more acceptance of the disease. They are willing to follow proper treatment. Further, they are primarily concerned about lab parameters. Lastly, they want to know if anything can be added to their treatment.

Risk communication

"Maybe this term is new to me, or I cannot comprehend it."

"Informally, we do risk communication. We do explain to the patient."

"It is crucial, but it is neglected… it is very practical."

"Communication is a time-consuming process… lack of time and the burden of patients that stops physicians from doing it with every patient."

"Many times we (clinicians) are not aware what should be done, kinds of words and what precise information…that sits in patients' brains, and which will make these patients more compliant".

Risk communication was a new term for all clinicians. However, they phrased it as "explaining the risks that diseases possess." They perceived it to be "very important" but felt that it was neglected; it could help patients understand the importance of the disease, treatment adherence, lifestyle modification, and ultimately prevent untimely deaths. It was neglected mainly due to "high patient load." They felt it was being practiced informally in the form of counseling. Clinicians were willing to incorporate it into consultations, as it would reduce the burden of the disease, but they questioned its feasibility. Furthermore, it was felt that it would help them with medico-legal aspects as well. They were open to the idea of formal training in risk communication methods.

## Discussion

Healthcare delivery is inherently a dynamic and bidirectional interaction between patients and physicians. Findings from the present study reinforce that patients enter clinical encounters with pre-existing ideas, beliefs, concerns, and expectations regarding their illness and treatment. These beliefs may be accurate or misconceived, and addressing them effectively is critical to ensuring appropriate disease management. Beyond diagnosis and treatment, patients often seek reassurance, clarity, and guidance on controlling their condition. One-sided communication is therefore unlikely to yield favourable outcomes, particularly for chronic conditions such as CVDs [15].

Clinicians in our study perceived CVDs as highly concerning because of their high prevalence, lifestyle-related etiology, and long-term complications. In contrast, patients frequently prioritised acute conditions and immediate symptoms, such as pain, over potential future cardiovascular consequences. This mismatch contributes to inadequate risk perception among patients. This gap is further widened by the incidental nature of diagnosis, as conditions such as hypertension and diabetes are often detected during routine encounters in asymptomatic and previously unaware individuals. Communicating long-term cardiovascular risk thus becomes particularly challenging when patients have not experienced overt illness or functional limitation, as engagement in risk discussions tends to be lower in the absence of physical symptoms [13]. This reduced engagement may contribute to poor lifestyle modification, as demonstrated by a Milan-based trial in which individuals with asymptomatic hypertension underestimated their risk and failed to change unhealthy behaviours [12]. As a result, clinicians are frequently required to introduce the diagnosis, communicate its implications, and motivate behavioural change simultaneously.

Better understanding of cardiovascular health has been associated with improved adherence to preventive measures and better outcomes. Accordingly, risk-related information must be conveyed in a manner that is credible, clear, and easy to understand [16] to minimise misinterpretation and reduce the likelihood of suboptimal treatment decisions. On the other hand, physicians in our study reported that patients often initially denied the diagnosis, questioned diagnostic measurements, feared future complications, or were apprehensive about lifelong treatment. Poor communication in these situations may further undermine trust in healthcare providers and contribute to anxiety and other adverse outcomes [13,17].

The concept of risk communication was new to all the clinicians interviewed in our study, but most of them reported practising it informally by explaining disease processes and complications. Although tools such as the Framingham risk score and Heart Age calculators have shown promise elsewhere [18-21], their feasibility and effectiveness were not evaluated in the present study and therefore warrant future research. A meta-analysis by Bakhit et al. also reported that communication of CVD risk improved the accuracy of risk perception and resulted in significant reductions in cardiovascular risk score at follow-up [22]. In our study, clinicians used diverse techniques to advise and motivate patients about medication and lifestyle changes concerning hypertension, diabetes, and CVD risk factors. While some communication was generic, other advice was personalised based on patients’ routines and sociodemographic characteristics. However, high patient load in Indian outpatient settings often limits consultation time, restricting the depth of communication. Clinicians reported attempting to deliver maximum relevant information within constrained time frames, frequently shortening discussions to accommodate patient volume. While clinicians expressed general satisfaction with the content of their communication, they emphasised the need for precise language and impactful messaging to influence patient behaviour effectively.

From a provider's perspective, balancing compassion with systemic pressures such as workload and time constraints remains a significant challenge [6]. Similar findings have been reported among Australian clinicians, where high patient load limited discussions on disease management and prevention [23]. Alternative approaches, such as the use of avatars, digital representations designed to promote engagement, have demonstrated improvements in risk perception and alignment between perceived risk and intention to seek care [24]. A few clinicians have also mentioned using ‘Scare tactic’ strategies in patients where CVD risk was high, or where patients had low motivation, emphasising potential future complications and adverse outcomes [6]. Whereas, a systematic review reported that several risk communication strategies, such as percentages, bar graphs, and icon arrays, have shown limited effectiveness in enhancing risk perceptions [13]. Evidence from India suggests considerable variability in provider practices regarding hypertension and diabetes management. To address these gaps, recent intervention protocols from Indian states such as Kerala and Tamil Nadu have also proposed structured, community-based strategies including peer support groups and health worker-led educational sessions to improve disease control through goal-based lifestyle changes, medication adherence, and active peer involvement using digital platforms such as WhatsApp (Meta Platforms, Inc., Menlo Park, California, United States) [25]. The UDAY programme in India similarly utilised tailored health promotion, technology-enabled education, and provider training to improve public awareness and disease management outcomes [26].

In our study, clinicians are very open to incorporating risk communication in their consultations. They are ready to welcome a formal method to communicate precise information that might enable the patient to take desirable actions to ensure appropriate treatment and reduce the overall burden of hypertension and diabetes. Evidence from a Houston-based survey further supports the effectiveness of collaborative and proactive patient-clinician communication, demonstrating improved hypertension control when shared decision-making and clinician-encouraged collaboration were prioritised [27]. 

This study represents a novel qualitative exploration of clinicians’ perspectives on communication with patients managing hypertension, diabetes, and CVDs in a tertiary care setting. It was conducted on all the clinicians among the healthcare providers, ranging from junior residents to consultants from various departments. However, the study has a few limitations. It was conducted in a single tertiary care institution with a small purposive sample, limiting the transferability of the findings. Data were derived solely from clinician interviews without triangulation or member checking and may therefore be subject to social desirability and interpretive bias. Additionally, the study explored perceptions and practices rather than the effectiveness of specific risk communication interventions.

## Conclusions

This study highlights the pivotal role of effective communication in the management of hypertension, diabetes, and CVDs. The study demonstrates that clinicians recognize the importance of communicating disease risk but face multiple barriers, including time constraints, language differences, and patient misconceptions. Future studies should evaluate structured risk communication interventions in Indian clinical settings before widespread implementation can be recommended. Strengthening clinicians’ capacity to convey risk effectively may enhance patient understanding, promote behavioural change, and ultimately reduce the burden of CVD.

## Appendices

Interview guide

Good morning/ afternoon sir/ madam! At the outset, I would like to convey my sincere thanks for accepting to participate in this study. As you have read in the PIS document, this interview will take approximately 40-45 minutes. I request you to provide your unstinted time during the interview. As you know, we would be recording the interview so that we can identify the important themes from the transcribed text later. I would like to assure you again that all the data collected from this session will be kept confidential and will be used for the purpose of research only. Your name or other personal details would not be used anywhere in the analysis or report writing. Let us start with your opinion on the most important diseases in our country.

1. Which are the diseases about which you are most concerned? Why do you say so? (Probe: most important diseases)

2. Which are the diseases about which other people are most concerned? Why do you say so?

Now we are going to focus on hypertension, diabetes, and other modifiable behavioral risk factors (tobacco, alcohol, unhealthy diet, physical activity) of CVDs. In this section, I will use the term ‘diseases’ for hypertension, diabetes, and CVDs together.

3. How common are these diseases and behavioral risk factors? (Probe: India, your state, district, locality)

4. On an average day, how many patients with these diseases visit you? What proportion of your total patient load forms these diseases? (Probe: total outpatient department (OPD) share, proportions of risk factors)

5. What are the local terms used by the patients for these diseases? What are the terms that you use while explaining these diseases?

6. What are the concerns that newly diagnosed patients discuss with you? (Probe: medication, diet, fear, apprehension, denial, reactions)

7. In your practice, how do you deal with the reactions of patients who have been newly diagnosed with hypertension, diabetes, or CVDs? 

8. What are the treatment goals you want your patients to achieve? (Probe: short and long term) What factors do you consider while deciding these targets?

9. How do you advise and motivate them about treatment adherence with medicines? (Probe: generic or personalized?) How do you personalize the advice?

10. How do you advise and motivate them about lifestyle modification? How do you personalize this advice? (Probe: advice changes with different patients)

11. What is the method of giving such advice? (Probe: verbal, print, written, video) Who does it? How easy or difficult is it for you to use this/these method(s)? Please explain or show us. (Probe: modify advice according to treatment goals)

12. Do you have enough time to explain the advice during the consultation? Why do you say so? (Probe: time spent in total consultation, discussion, explaining the disease, treatment, etc)

13. How easy or difficult is it for patients to follow the advice? (Probe: taking medicines, change in lifestyle)

14. How relevant are the issues of patient concerns, lifestyle, and treatment adherence to you? What effect do these issues have on the overall health status of patients? (Probe: premature deaths, premature acute coronary syndrome (ACS), cost of care, crowds in hospital, etc.)

15. How do previously diagnosed patients differ in terms of concerns and other behaviors? Why do you think the previously diagnosed patients behave differently? (Probe: duration of diagnosis, age, sex, etc.)

16. Do you change your advice, motivation, and non-pharmacological treatment strategy with previously diagnosed patients? If yes, how and why? (Probe: Clinical vignette, if the clinician agrees to show the risk communication method s/he practices in routine patient care) 

We have finished our discussion of how you deal with patients having hypertension, diabetes, and other modifiable behavioral risk factors (tobacco, alcohol, unhealthy diet, physical activity) of CVDs. We would like to discuss “risk communication” now. 

17. What is your understanding of risk communication?

18. What is your experience with risk communication in your practice? (Probe: use/practice, importance, practicality, willingness among clinicians and patients)

Before finishing, I am going to briefly explain the concept of ‘risk communication’. In many countries, reduction in premature death is one of the prominent treatment goals. Clinicians often use methods of risk communication to inform about the risk of premature death or other serious complications. It usually takes three to four minutes depending on the method used. Subsequent visits have been found to be brief, and patients are more compliant with medicine and lifestyle changes.

19. What are your opinions about formal training in methods of risk communication? (Probe: willingness, importance, methods, trainer)

20. Anything else you would like to add regarding the study topic?
